# Heterogeneous Rate Constant for Amorphous Silica Nanoparticle Adsorption on Phospholipid Monolayers

**DOI:** 10.1021/acs.langmuir.1c03155

**Published:** 2022-04-26

**Authors:** Alex Vakurov, Rik Drummond-Brydson, Nicola William, Didem Sanver, Neus Bastús, Oscar H. Moriones, V. Puntes, Andrew L. Nelson

**Affiliations:** † School of Chemistry, University of Leeds, Leeds LS2 9JT, U.K.; ‡ School of Chemical and Process Engineering, University of Leeds, Leeds LS2 9JT, U.K.; § Department of Food Engineering, Faculty of Engineering, Necmettin Erbakan University, Konya 42050, Turkey; ∥ Catalan Institute of Nanoscience and Nanotechnology (ICN2), CSIC, The Barcelona Institute of Science and Technology, Campus UAB, Bellaterra, Barcelona 08193, Spain; ⊥ Universitat Autònoma de Barcelona (UAB), Campus UAB, Bellaterra, Barcelona 08193, Spain; # Fundacio Hospital Universitari Vall D’Hebron - Institut De Recerca, Passeig Vall D Hebron, 119-129, Barcelona 08035, Spain; ∇ ICREA, Pg. Lluıs Companys 23, Barcelona 08010, Spain

## Abstract

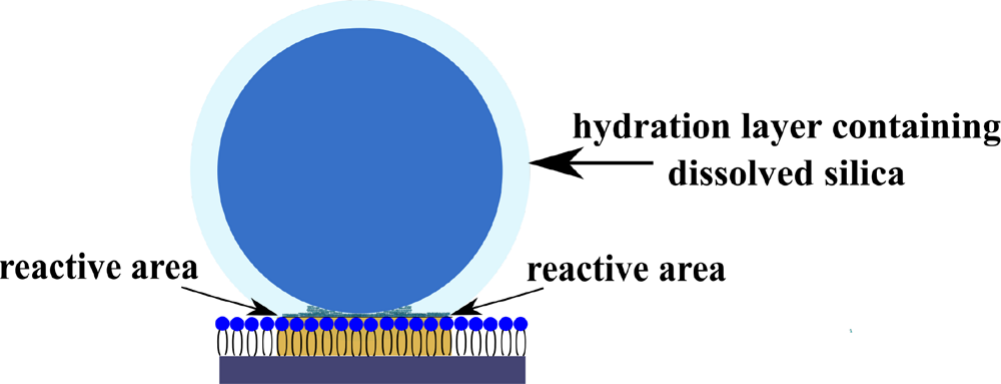

The interaction of amorphous silica nanoparticles with phospholipid monolayers and bilayers has received a great deal of interest in recent years and is of importance for assessing potential cellular toxicity of such species, whether natural or synthesized for the purpose of nanomedical drug delivery and other applications. This present communication studies the rate of silica nanoparticle adsorption on to phospholipid monolayers in order to extract a heterogeneous rate constant from the data. This rate constant relates to the initial rate of growth of an adsorbed layer of nanoparticles as SiO_2_ on a unit area of the monolayer surface from unit concentration in dispersion. Experiments were carried out using the system of dioleoyl phosphatidylcholine (DOPC) monolayers deposited on Pt/Hg electrodes in a flow cell. Additional studies were carried out on the interaction of soluble silica with these layers. Results show that the rate constant is effectively constant with respect to silica nanoparticle size. This is interpreted as indicating that the interaction of hydrated SiO_2_ molecular species with phospholipid polar groups is the molecular initiating event (MIE) defined as the initial interaction of the silica particle surface with the phospholipid layer surface promoting the adsorption of silica nanoparticles on DOPC. The conclusion is consistent with the observed significant interaction of soluble SiO_2_ with the DOPC layer and the established properties of the silica–water interface.

## Introduction

The interaction of silica nanoparticles with biological systems, in general, has achieved an increasing amount of interest over the last decade.^1−3^ At a more specific cellular level, the contact of some types of silica particles with the membrane of cells, particularly erythrocytes, is known to cause its rupture and cellular death.^4−7^ In addition, because silica nanoparticles have been extensively proposed as carriers in nanomedicine,^8^ assessing their potential toxicity is of utmost importance and a primary aim of fundamental research. Furthermore, toxicity is strongly dependent on the type of silica polymorph and its precise surface chemistry. For instance, the US Food and Drug Administration (FDA) regards amorphous silica as generally recognized as safe (GRAS), whereas crystalline silica nanoparticles can be cytotoxic.^5^ However, so-called fumed silica (amorphous silica nanoparticles produced by gas-phase pyrolysis) does induce cytolysis and is also toxic to certain cellular strains. In contrast, colloidal silica such as Stoeber silica^9^ and so-called mesoporous silica nanoparticles are much less harmful to eukaryotic cells and result in significantly less cytolysis than fumed silica.^4,7^ Notably, the sol–gel synthesis of Stoeber silica nanoparticles happens via a monomer-addition process similar to naturally occurring silica biomineralization.^9,10^ As a result, owing to the fact that the molecular initiating event (MIE) involved in the biological activity of silica nanoparticles often involves the interaction of the nanoparticle with the cell membrane, many studies have investigated the interaction of silica particles and silica surfaces with phospholipid membrane models^11−22^ because these are the underlying backbone of the biological membrane. However, in spite of the fact that silica particles have been shown to interact strongly with phospholipid membranes, the precise mechanism of interaction and the species involved therein remain uncertain.

Pera and coworkers^11^ studied the coverage and disruption of phospholipid membranes with silica and titania nanoparticles. Their approach was comprehensive in that they used both vesicles and supported lipid bilayers to implement their study in the same way as completed in a previous study.^12^ Their studies followed two studies^13,14^ published by this laboratory, which conclusively showed the adsorption of silica nanoparticles on, and disruption of, phospholipid monolayers and bilayers. Since then, there have been several studies on this subject^15−19^ in addition to reports on the interaction of silica surfaces with lipid membranes^20−22^ in general. One of the main conclusions of Pera et al.^11^ was that electrostatic forces play a key role in the interaction. To elucidate the interaction mechanism, they looked at the rate of adsorption of the silica nanoparticles on to the supported phospholipid bilayers. However, Pera’s results cannot be directly compared with the results in this study because different experimental setups were used and different hypotheses were pursued. On the other hand, fundamentally identical systems are being studied employing the same silica particles (Ludox) and the same lipids (DOPC) in the supported conformation. Nonetheless, Pera et al. extracted no absolute rate constants from their data and speculated on a barrier to the adsorption rate. The lack of conclusions from their rate experiments in their excellent piece of work has prompted this laboratory to revisit this laboratory’s adsorption model^13^ and to significantly extend the experiments together with the analysis. From working with dioleoyl phosphatidylcholine (DOPC) and dimyristoyl PC (DMPC) monolayers and bilayers, it had been concluded that van der Waals forces were responsible for the interaction.^13,14^ This conclusion had been arrived at because the analysis showed the presence of a “reaction layer” (*h*) on the surface of the particle of about 3.2 nm in thickness, above which no interaction between the silica nanoparticle and the lipid monolayer occurs. Subsequent studies cited above^15−22^ have characterized a similar interaction between lipid membranes and silica surfaces and hypothesized on the forces responsible for the interaction, ranging from electrostatic to van der Waals to hydrogen bonding and/or a combination of all three. This presented a double motivation for this laboratory to return to the work in more detail and assess if any new results and subsequent new analysis thereof could clarify the mechanisms of silica nanoparticle/lipid membrane interaction.

Preliminary results from this laboratory showed that the adsorption rate of amorphous silica nanoparticles on Hg-supported DOPC monolayers is remarkably well behaved.^13^ This is because the experiments were carried out in a flow cell where the sample flow rate was 10 cm^3^ per minute in a flow cell with a volume of 0.75 cm^3^. This minimizes any diffusional control of the adsorption rate. The adsorption rate, which is measured as suppression in the capacitance current peak in the rapid cyclic voltammogram (RCV) of the DOPC layer, was found to be linearly related to the concentration of SiO_2_ as mmol dm^–3^ in aqueous dispersion. Accordingly, the adsorption rate was normalized to the SiO_2_ mmol dispersion concentration, and this normalized rate (*k*’) seemed to vary linearly and inversely with the particle size.^13^ The objective of the present study, therefore, was to comprehensively extend the adsorption rate experiments and analysis to larger particle sizes to derive a heterogeneous rate constant for amorphous SiO_2_ nanoparticle adsorption on the DOPC monolayer. From the dependence of this rate constant on the particle size, further insight into the adsorption mechanism of silica particles on phospholipid layers might be obtained.

An additional aim of this study was to use the derived rate constant in physiologically based pharmacokinetic (PBPK) models^23^ to define the first step or molecular initiating event in the uptake of SiO_2_ particles by the cell membrane,^24^ leading to the cell membrane’s ultimate damage. Additional experiments were also carried out on the effect of the silica nanoparticle dispersion supernatant on the DOPC layer. In the text, silica nanoparticles and silica surfaces are referred to as such, whilst molecular silica and associated derived species are referred to as SiO_2_.

## Materials and Methods

The silica dispersions used for the adsorption rate experiments were sourced exactly as described previously.^13,14^ (see Table S1 in the Supporting Information). The nanoparticle hydrodynamic size, as determined by dynamic light scattering (DLS), was used in all calculations. These dispersions were also purified by gel filtration immediately prior to the experiment. The dispersions remained stable and not agglomerated throughout each rate experiment (see Figure S1 in the Supporting Information). DOPC was obtained to prepare the monolayers, as detailed in earlier studies.^25−27^ The equipment and methods used were as described earlier.^13,25−27^ In principle, the adsorption of silica particles from a static and flowing dispersion onto a supported DOPC monolayer was measured by RCV at 40 V s^–1^ with a voltage excursion from −0.4 to −1.2 V, as described previously.^13,26^ The monolayer was deposited on a fabricated Pt/Hg electrode positioned in a flow cell. The silica dispersion was in 0.1 mol dm^–3^ KCl buffered with 0.01 mol dm^–3^ phosphate buffer at pH 7.5, a pH at which all rate experiments in this study were carried out. The adsorption of the particles on the DOPC affected the depression of two capacitance current peaks 1 and 2 (see Figure S2 in the Supporting Information), where the extent of peak depression relates to the particle coverage of the layer and the size of particles. This peak depression is a result^13^ of the adsorption of the particles onto the lipid layer surface, “freezing” the layer^14^ and impeding the phospholipid lipid reorientations. The particle charge had some effect on this adsorption process because when the pH of the particle dispersion was taken to 4, which is closer to the PZC of 2.8^28^ of the silica particles lowering their negative charge, the interaction between particles and DOPC increased slightly^13^ (see Figure S3 in the Supporting Information). It has been shown^13^ that the current peak 2 at more negative potentials was the most exact indicator of particle coverage on the monolayer. The measurement of this peak height was taken from the height of the peak to the RCV baseline, which in the case of complete peak suppression, is shown in Figure S2 in the Supporting Information.

**Figure 1:**
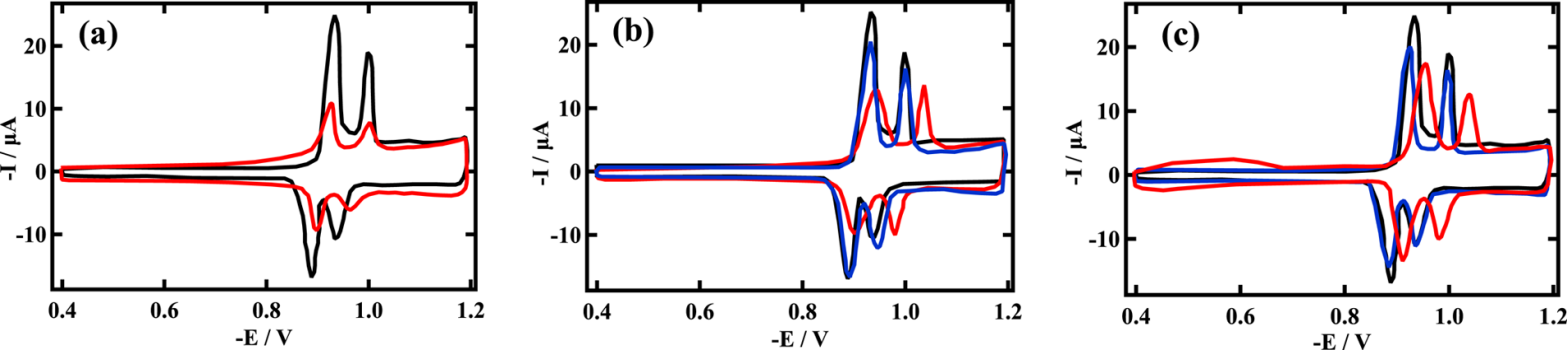
RCV of DOPC on a Pt/Hg electrode recorded at 40 V s^–1^ control PBS (black) and after; (a) exposure in flow to 0.169 mol dm^–3^ (SiO_2_) 17.5 nm radius Stoeber^10^ synthesized silica nanoparticles in Milli Q water at pH 8.4 (red); (b) exposure to silica dispersion supernatant from (a) (red) and 0.06 mol dm^–3^ (TiO_2_) titanium dioxide dispersion (2.5 to 5 nm radius) supernatant (blue); and (c) recovery from interaction with silica dispersion in (a) (red) and with silica dispersion supernatant in (b) (blue).

The adsorption can be followed in “real-time” by continuing the RCV monitoring during the adsorption process. In this manner, the peak current plotted against time gives a measure of the extent of adsorption versus time. The initial slope of this plot characterizes the initial rate of adsorption, which decreases as the DOPC surface becomes occupied with particles. These effects are shown very clearly in Figure 1B of a previous study^13^ (Figure S3 in the Supporting Information), where preliminary studies of adsorption rate were carried out by assessing the slope of the plot between times 0 and 50 s. A parameter, *k*’, is obtained, which is the initial slope of this plot divided by the silica concentration as SiO_2_ and is defined as the normalized rate with units μA mol^–1^ dm^3^ s^–1^. In this present study, extensive adsorption rate determinations were carried out precisely this way for the full range of particle sizes (6.75 to 85 nm radius) and SiO_2_ concentrations (0–12 mmol dm^–3^). The relation between the rate of development of the adsorbed silica layer as defined by the slope of the plot and the relative peak height at equilibrium on the Y axis is shown very clearly in Figure S3 in the Supporting Information and Figure 1B,C of a previous study.^13^ We define equilibrium adsorption on these figures as that attained when the rate decreases to a constant value equivalent to that of the control, as shown in Figure 1B,C of a previous study^13^ and Figure S3 (curves a, and *a*_1_ and *a*_2_ respectively) in the Supporting Information and is directly linked to the complete coverage of the DOPC layer with nanoparticles, as evidenced by scanning electron microscopy (SEM) images (Figure S4a–c in the Supporting Information). Indeed, in the previous study,^13^ this equilibrium peak suppression was plotted against the particle size (Figure S5 in the Supporting Information), and the equilibrium adsorption model as eq 6 in the previous study^13^ was developed from this.

In order to investigate the interaction of soluble SiO_2_, as opposed to silica particles, with DOPC layers, 17.5 nm radius particle dispersions (polydispersity index (PDI) = 0.11) in MilliQ water were used. These dispersions had a pH of 8.5 because the silica nanoparticles were synthesized using the Stoeber method^9^ and contained traces of ammonia. Ammonia traces had no effect on the agglomeration of the particles because the transmission electron microscopy (TEM) image and the DLS plot showed single particles in the dispersion (see Figures S6 and S7 in the Supporting Information). The concentration of SiO_2_ in the dispersion was 0.169 mol dm^–3^. The dissolved SiO_2_ was separated from the dispersion by centrifugation at 10,000 to 15,000 *g*, relative centrifugal force (RCF), for 20 min and collecting the supernatant. Using small Eppendorf 1.5 mL centrifugation tubes, centrifugation is efficient and sediments almost 100% of the particulate silica. Evidence for this is provided by the fact that the derived mean count rate of 100–120 counts per second from DLS measurement and the optical density at 360 nm of the supernatant resembled that of MilliQ water. These values of the silica dispersion are very much larger (see Figure S8 in the Supporting Information). The sedimented silica was resuspended and stored in MilliQ water. These dispersions were not gel-filtered prior to screening and contained dissolved SiO_2_ in equilibrium with the particulate silica. Both the dispersions and centrifuged supernatant were screened for silica nanoparticles and SiO_2_ interaction with DOPC using the flow system platform, as described in a recent study.^29^ A supernatant of 0.06 mol dm^–3^ (TiO_2_) titania nanoparticle dispersions, prepared from the same source and similarly centrifuged, was also screened as a control. Monolayer recovery experiments were carried out on the interactions of both the dispersion and the supernatant with the DOPC monolayer by introducing control phosphate-buffered saline (PBS) into the flow sensor module in place of the dispersion or supernatant in PBS. Accordingly, following the interaction experiment, PBS was flushed through the flow cell for 400 s with continuous RCV cycling from −0.4 to −1.2 V to allow for and to monitor any recovery of the DOPC layer’s initial structure to occur. These experiments have been carried out previously to follow the recovery of DOPC layers, following interaction with organic compounds.^29,30^ The forced convection (10 cm^3^ min^–1^) in the flow cell (0.1 cm × 7.5 cm^2^) minimizes any diffusional control of adsorption and means that adsorption is predominantly under kinetic control,^31^ as justifiably assumed throughout this study.

### Analysis

From the previous study,^13^ a specific “reactive area” is the area of the silica particle that interacts with the DOPC layer and extends from the physical contact of the silica particle with the lipid layer up to the “reaction layer” thickness and is defined by the numerator of eq 5 multiplied by, in this instance, the number of particles in 1 mmol. ***A*** can therefore be expressed with units of cm^2^ mmol^–1^ as follows:
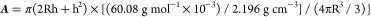
1where R is the particle radius in cm, h is the “reaction layer” thickness (=3.23 × 10^–7^ cm), 60.08 g mol^–1^ is the molecular mass of SiO_2_, and 2.196 g cm^–3^ density of amorphous silica. It should be noted that when eq 6 in a previous study^13^ equals zero, all the particles on the DOPC monolayer are close-packed such that their “reactive areas” are confluent. At smaller particle sizes eq 6^13^ is forced to equal zero, and the value of the “reaction layer” thickness, h, and the available “reactive area” become smaller.

There are two methods for calculating the heterogeneous rate constant for silica particle adsorption on DOPC: the “bottom-up” and the “top-down” procedures, respectively. The “bottom-up” approach considers the adsorption of SiO_2_ molecular species on DOPC layers, whereas the “top-down” approach considers the adsorption of silica nanoparticles on DOPC layers. The heterogeneous rate constant described in this study is the rate of growth of an adsorbed layer (molecules or nanoparticles) per unit area divided by unit bulk solution or dispersion concentration of those molecules or nanoparticles. Accordingly, the division of the normalized rate (*k*’) by the current peak depression corresponding to the full coverage of particles on the DOPC gives the fraction of full coverage per bulk unit solution concentration growing as an adsorbed layer on the DOPC in 1 s. If this value is multiplied by the full coverage (=reciprocal of area per mmol), a value for the growth rate of the adsorbed layer per unit area of the surface from unit solution/dispersion concentration is obtained.

In the “top-down” approach, the same arguments are used as above, but instead, the adsorption of nanoparticles is considered. In this case, the normalized rate (*k*’) is converted to a heterogeneous adsorption rate constant (*k*_2_ and k_2_) by dividing it by the maximum depression of the capacitance current peak specific to that particle size (Figure S2 or Figure 1A^13^) and multiplied by the millimolar close-packed nanoparticle coverage on the DOPC surface. The maximum experimental depression of the capacitance peak current specific to a particular particle size has been used to calculate *k*_2_. The maximum depression of the capacitance peak current particular to a nanoparticle size can also be estimated from eq 6,^13^ which closely fits the data. The surface area (SA) on a surface in cm^2^ occupied by a mmol of nanoparticles^13^ is 6.02 × 10^20^ × 2 × √3 × R^2^. This area represents the total area available for binding of the nanoparticles, and the reciprocal of this number is the close-packed nanoparticle coverage per cm^2^. A factor '*v*' or 'v' (depending on how it is calculated) can be identified, which is the maximum depression of the capacitance peak current specific to a particle size multiplied by the SA and in the case where the maximum depression of the capacitance peak current particular to a particle size is obtained from the experiment, *v*, can be directly calculated. v can also be estimated from eq 6,^13^ where the maximum depression of the capacitance peak specific to the particle size is 32.1 μA × π(2Rh + h^2^)/(2 × √3 × R^2^). Therefore v = 32.1 μA × π(2Rh + h^2^) × 6.02 × 10^20^. This is used in the calculation of k_2_. The equations for *k*_2_ and k_2_ are as follows:
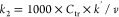
2
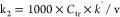
3where, because *k*’ as the numerator is normalized to 1 mmol of molecules (per dm^3^) and the denominator ( *v* or v) is normalized to a millimole of particles (per cm^2^), it was necessary to multiply the numerator by the number of SiO_2_ molecules in one silica nanoparticle (*C*_tr_) and, by 1000 to bring all units to cm and by *C*_tr_= 6.02 × 10^23^ × 4/3πR^3^ × 2.196 g cm^–3^/60.08 g mole^–1^.

## Results and Discussion

Figure S2 in the Supporting Information displays voltammograms of the interaction of a gel permeation-purified silica nanoparticle dispersion; Figure 1a shows a MilliQ water-equilibrated silica dispersion, and Figure 1b,its supernatant with DOPC monolayers. Also displayed in Figure 1b is the interaction of the supernatant from titania nanoparticle dispersions as a negative control. Figure 1c shows the recovery RCVs following silica nanoparticle dispersion and its supernatant’s interaction with DOPC and subsequent flushing with PBS. Figure S2 is reproduced from Figure 1A,^13^ including an additional RCV plot, and is displayed to clarify the RCV response to the silica nanoparticle/DOPC interaction. Capacitance current peaks 1 and 2 are labeled thereon. RCVs in Figure 1a–c were obtained in the current work. Because separate platforms were used to obtain Figures S2 and 1a–c, the current values in Figures S2 and 1a–c differ, but the RCV profiles are similar. Indeed, the RCV profiles following interactions are interesting because they exhibit similarity in terms of the interaction of amorphous and Stoeber^9^ silica particles with the DOPC layer. Although two very different dispersion concentrations of silica were used, the total coverage of the DOPC layer with nanoparticles was maintained. Particularly evident in Figure 1b is the influence of the supernatant interaction with the DOPC layer, which elicits a similar but not identical interaction to that of the silica nanoparticle dispersion. Significantly, as shown in Figure 1b, the capacitance current peaks are shifted to negative potentials. The almost insignificant effect of the titania nanoparticle dispersion supernatant on the RCV profile (as a negative control) establishes the silica nanoparticle supernatant dispersion interaction as significant.

Of additional interest is the effect of the recovery experiments shown in Figure 1c. Following the interaction of the silica nanoparticle dispersion supernatant with the DOPC layer and the DOPC layer’s recovery through flushing with PBS, the RCV capacitance current peaks are close to resembling those of the control. On the other hand, following the interaction of the silica nanoparticle dispersion with the DOPC layer and the DOPC layer’s recovery, the RCV profile shows a more significant departure from that of the control, in particular, an increase in the baseline capacitance current at more positive potentials. Intuitively, molecular SiO_2_ presumably present in the supernatant can be more readily removed from DOPC into solution than adsorbed silica nanoparticles can be removed into the dispersion phase.

**Figure 2:**
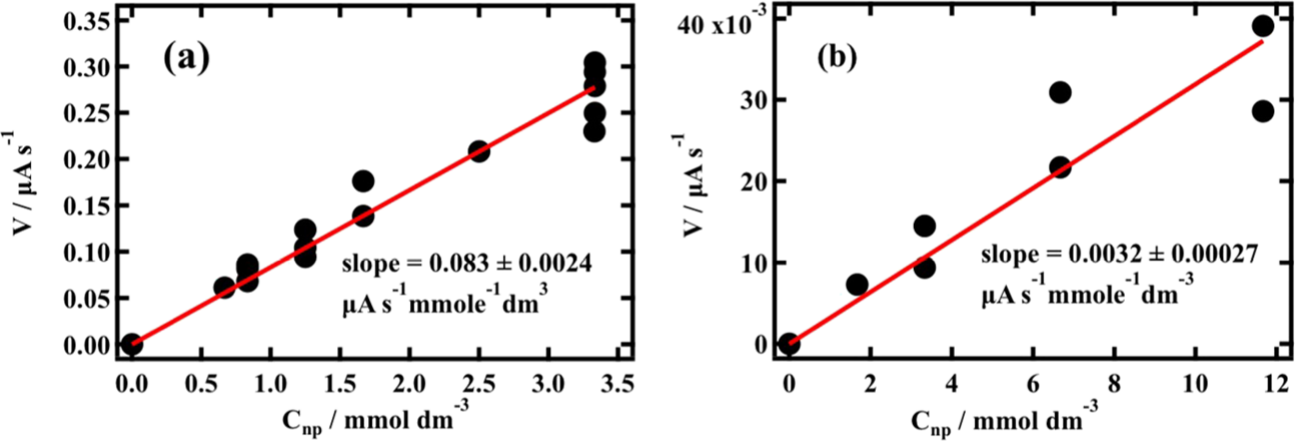
Plots of the rate of decrease in the height of capacitance current peak 2 height (*V*) versus silica nanoparticle bulk concentration as mmol dm^–3^ SiO_2_ (C_np_) derived from RCVs of DOPC on Pt/Hg exposed to (a) 15.05 nm radius and (b) 86.05 nm radius nanoparticles.

Figure 2a,b shows representative plots of the rate of adsorption (*V*) as measured by the initial rate of decrease in capacitance current peak 2 height in μA s^–1^ plotted against silica particle concentration expressed as mmol dm^–3^ of SiO_2_ (C_np_) for two respective silica nanoparticle sizes (see Figure S9 in the Supporting Information for all rate versus silica concentration plots obtained in this study). The rates of decrease in the capacitance current peak height in units μA s^–1^, as measured using the method shown in Figure S3 in the Supporting Information, are inversely related to the initial rate of growth of an adsorbed silica layer on the DOPC monolayer. All *V* versus C_np_ plots measured are linear.

**Figure 3:**
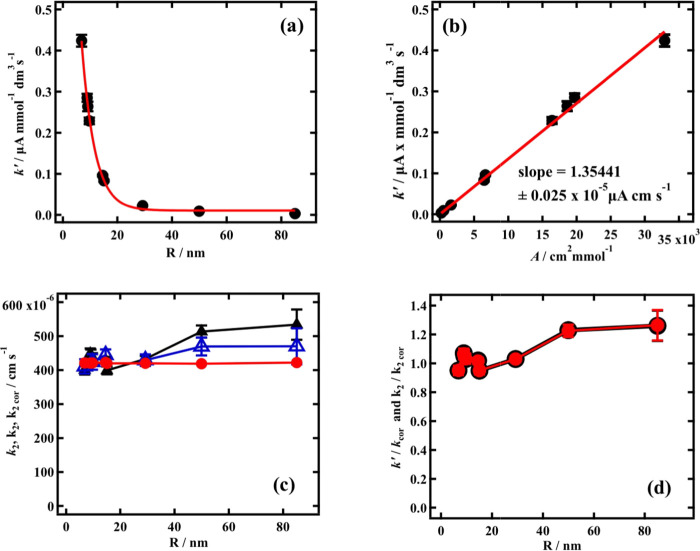
Plots of (a) “normalized” rate (*k*’) derived from slopes of *V* versus C_np_ plots versus silica nanoparticle radius (R); (b) *k*’ versus the “reactive area” of silica nanoparticles (***A***) specific to each value of R; (c) heterogeneous rate constants, k_2_ (filled black triangles), *k*_2_ (blue triangles), and k_2cor_ (filled red circles) for silica adsorption versus R; and (d) *k*’/*k*’_cor_ (red-filled circles) and k_2_/k_2cor_ (black circles) versus R. Errors in (c) and (d) are propagated from the errors of *k*’ estimation, and for *k*_2_, they include the capacitance current peak measurement error.

The value of the normalized rate, *k*’, is defined as the slope of the plots of *V* versus SiO_2_ concentration (C_np_), and its standard deviation (SD) is extracted from the linear fit to the plots, as shown in Figure 2a,b. An extension of the *k*’ measurement to larger particle sizes compared to the preliminary measurements carried out previously^13^ shows that the relationship between *k*’ and the particle size is not linear, as originally derived for small particle sizes but actually exponential (see Figure 3a). The interesting feature of the normalized adsorption rate is that if it is plotted against the specific “reactive area” (***A***) available for adsorption, a linear relationship is obtained with *R*^2^ = 0.99 (as shown in Figure 3b).

Conceptually, the slope of the plot in Figure 3b represents the normalized rate, *k*’, at a specific nanoparticle size divided by the “reactive area” at the same size per mmol SiO_2_, a ratio which is constant irrespective of the particle size (Figure 2b). It is to be noted that the reciprocal of the “reactive area” is the surface coverage, of total SiO_2_ mmoles in the particles, on a cm^2^ of “reactive area,” where, by definition, every silica surface SiO_2_ unit interacts with the DOPC. Accordingly, as the particles become larger, this coverage becomes smaller.^13^ At the same time, it is known^13^ that a close-packed coverage of the DOPC monolayer with silica nanoparticles of such size that their “reactive areas” are confluent will lead to the total depression to the baseline of the capacitance current peak 2 of 32.1 μA.^13^ A RCV reflecting adsorption of the smallest SiO_2_ particles on the DOPC layer is also displayed in Figure S2 in the Supporting Information. It is noted that the capacitance current peak 2 is almost totally suppressed to be continuous with the baseline capacitance current. SEM images in the previous study confirmed complete close-packed coverage of the silica layer at equilibrium adsorption (see Figure S4 in the Supporting Information). This is in contrast to Pera et al.^11^ results, which show that only incomplete coverage (∼30%) of silica particles on pure DOPC monolayers is obtained. In this study, however, whenever a unit area of lipid layer covered by a “reactive area” is considered, there is a total interaction of the SiO_2_ units with that unit area. Therefore, the silica particles could cover a much larger area than the unit area, but the “reactive area” will always occupy the same unit area. Accordingly, the ratio between total SiO_2_ coverage per specific “reactive area” becomes smaller with larger particle size, and the *normalized* adsorption rate becomes smaller, as seen in Figure 3b.

The “bottom-up” approach takes the slope of 1.36 ± 0.025 × 10^–5^ μA cm s^–1^, as shown in Figure 3b, and divides it by the maximum current depression (32.1 μA). The result, using the above reasoning, is the heterogeneous rate constant of SiO_2_ adsorption (k_1_) of 4.22 ± 0.077 × 10^–4^ cm s^–1^. The constancy of the slope shown in Figure 3b shows that k_1_ is independent of the silica particle size because each point shown in Figure 3b represents a different particle size. The equation for the calculation can be summarized as follows:
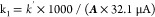
4where *k*’ is multiplied by 1000 because it has units of mmol dm^–3^ and requires to be converted to mmol cm^–3^, and ***A*** comes from eq 1.

Results for *k*_2_ and k_2_ estimated using the “top-down” approach can be observed in Figure 3c. It is seen that k_2_ =4.48 ± 0.44 × 10^–4^ cm s^–1^ and is generally constant with respect to the silica particle size when the particle size is small but shows a small increase at the largest particle sizes. When the experimental maximum decrease in capacitance current peak height is used in the rate constant estimation, the increase in *k*_2_ =4.35 ± 0.34 × 10^–4^ cm s^–1^ with the particle size is less significant. The apparent increase in k_2_ can be minimized by multiplying the best fit slope value from the *k*’ vs *A* plot (Figure 3b) with the “reactive area” specific to each particle radius and substituting this value termed *k*’_cor_ into eq 3. The adjusted value of k_2_ expressed as k_2cor_ is now effectively constant with respect to the particle size with a mean value of 4.22 ± 0.01 × 10^–4^ cm s^–1^, which is not significantly different from the value of k_1_ estimated from the slope of *k*’ versus ***A***. In fact, the effective equality of the “bottom-up” k_1_ value to the “top-down” k_2cor_ value comes from the fact that the algebraic treatment of the data in both approaches is identical, as shown by expanding and comparing eqs 3 and 4, which has been exemplified in the Supporting Information (eqs S4 and S9, respectively). This arises from the relation between the depression of the capacitance current peak and the particle size being underwritten by the way the particles of increasing sizes interact with the DOPC layer through the “reaction layer” (see eq 6^13^).

It is instructive now to assess whether there is any significance in the increase of k_2_ with respect to the particle size. To do this, the deviations of the experimental values of *k*’ from the best fit slope line of the *k*’ versus ***A*** plot were estimated from the value of *k*’ divided by the value of *k’*_cor_. These values are displayed in Figure 3d together with the k_2_/k_2cor_ ratios corresponding to each particle size. The *k*’ measurement errors are also shown in the diagram and are divided by *k*’_cor_ for a given particle size. Similarly, the *k*’ measurement errors that are propagated through in the calculation of k_2_ are divided by k_2cor_ and displayed in the same diagram. The plots of *k*’/*k*’_cor_ and k_2_/k_2cor_ coincide as expected. It can be seen from this that the increase in k_2_ with the particle size exactly mirrors the positive deviation of *k*’ from the linear best fit line of *k*’ versus ***A***. This deviation is more significant at the larger particle sizes and represents an adsorption rate overestimation error at low values. Indeed, the increase in k_2_ is only marginally outside the *k*’ measurement error and is thus barely significant.

Several points can be concluded from this analysis. First, the evidence points to the same rate-determining step controlling the baseline silica adsorption which is almost independent of the silica particle size. This can occur if molecular SiO_2_ and small hydrated SiO_2_ species promote the adsorption of particulate silica onto the lipid monolayers. Because molecular SiO_2_ and small hydrated SiO_2_ species such as silicic acid, SiO_2_ polymorphs, and silanol groups have considerable H-bonding potential,^32^ H-bonding between the phospholipid polar groups, in particular, the hydrated phosphate moieties and the hydrated SiO_2_ species will play a large part in adsorption.^21,22^ Second, the meaning of the “reaction layer” thickness has to be re-examined. Previously, it was thought that this was the maximum distance over which van der Waals forces could operate to facilitate adsorption. However, the rate constant data show that it is unlikely that van der Waals forces are implicated as a main driving force in adsorption. Van der Waals forces do vary inversely with the particle size,^33,34^ and if they promoted adsorption, a negative dependence of the rate constant on the particle size would be expected. In addition, the particle geometry plays a key role in the adhesion force between particles decreasing in order for plates, cylinders, and spheres of a given mass when van der Waals forces are considered.^35^ In this study, the particle geometry in the form of curvature is changed by a factor of >10, but clearly, this has no significant effect on the adsorption rate constant. The extent of the significance of VdW forces in the adsorption of amorphous SiO_2_ onto DOPC monolayers could be investigated directly through force-distance measurements using an atomic force microscopy (AFM) probe coated in silica in close proximity to a supported DOPC layer. A similar study has been carried out elsewhere.^36^ This laboratory has investigated the behavior of several other classes of nanoparticles with respect to their interaction with phospholipid layers within the platform set up used in this study including -TiO_2_^27^ and ZnO,^37^ where their polarizability compared to SiO_2_ is more or less similar. If VdW forces were the main factor promoting adsorption, their adsorption behavior would be expected to be more or less similar and not greatly different, except showing a particle size dependence. In fact, SiO_2_ adsorption on DOPC is very different from that of other main classes of nanoparticles in that it fits a straightforward equilibrium model.^13^

Where both physical and chemical forces are responsible for particle adsorption on a surface, the rate constant of the adsorption of nanoparticles can be dependent on their particle size in various ways.^38^ The facilitation of particle adsorption by molecular SiO_2_ and more complex hydrated SiO_2_ species throws new light on the “reaction layer” thickness and the “reactive area” concept.

Recent observations have indicated that amorphous silica is covered by a layer of hydrated SiO_2_ in various forms^39−44^ and that the adsorption of lipid vesicles on amorphous silica surfaces is in fact promoted by water molecules associated with the surface in layers up to 2.5 nm thick.^22^ This aforementioned study^22^ concluded that in the adsorption of lipid vesicles on a silica surface, there were two thermodynamically stable adsorption sites characterized by different widths of the water layer between the membrane and the substrate. It is reasonable to conclude that it is simply a hydrated layer promoting the adsorption of silica onto DOPC layers. Recent evidence^45^ has shown that TiO_2_ nanoparticles are also surrounded by a hydrated layer which, by the previous argument, could suggest that the interaction of TiO_2_ with the phospholipid layer would be similar to that of SiO_2_. In fact, the interaction of TiO_2_ nanoparticles with lipid layers is quite different.^27^ We believe that one of the reasons for this difference is that SiO_2_ dissolves slowly in water, and TiO_2_ is highly insoluble in water.

A series of investigations of ZnO,^37^ CdS,^46^ and Au and Ag^47^ nanoparticle interactions with the phospholipid layer shows that it is primarily the nanoparticle surface properties and coating that drive the interactions. This leads us to think that the “reaction layer” thickness is composed of an interphase between amorphous silica and the electrolyte consisting of a progressively hydrated layer of loose SiO_2_ molecules and more complex hydrated SiO_2_ species, which promote the silica/DOPC monolayer interaction. The evidence that amorphous silica dissolves in water and the electrolyte to 0.012%^48,49^ or 0.002 mol dm^–3^ in solution supports this idea. Many further studies^50−53^ have looked at the rate of silica dissolution, which can be of the order of hours^53^ and which increases markedly with solution pH.^53^ Soluble SiO_2_ has been shown to play a part in the toxicology of particulate silica to the lung tissue.^53^ A recent study has indeed confirmed the existence of surface-bound water stabilized by the silanol-rich groups on amorphous silica surfaces.^54^ This has been preceded by a study which showed that a silica gel-like structure of 1–2 nm thickness of silicic acid and silanol grows on hydrophilic silica surfaces.^55^ This interfacial “reaction layer” might also diffuse more within the phospholipid polar group region. A recent model of the aqueous SiO_2_ biomembrane interphase has predicted that Si(OH)_4_ and (HO)_3_SiO^–^ species can penetrate deep into the lipid membrane. In addition, hydrated SiO_2_ clusters are able to spontaneously enter deep into the region occupied by the lipid heads. There, they form a strong network of hydrogen bonds with the negatively charged phosphate groups^56^ and possibly also with the cationic polar heads.

This study has shown that soluble SiO_2_ is active on the DOPC surface (see Figure 1c). The silica dispersion’s supernatant interaction is similar to that of the silica dispersion but somewhat lessened, and the capacitance current peaks are shifted to more negative potentials. It has already been shown^48,49^ that at the solution pH 8.4 of the silica dispersion, a proportion of the solubilized SiO_2_ will exist as both the Si(OH)_4_ molecular species and the (HO)_3_SiO^–^ ion as well as more complex condensed species.^48^ The adsorption of the negatively charged (HO)_3_SiO^–^ on the DOPC layer can give rise to the negative potential shift of the capacitance peaks.^57^ It is appreciated that a small amount of fine colloidal silica will have escaped the centrifugation process in this study. On the other hand, DLS and UV absorption evidence indicates that the supernatant more nearly resembles MilliQ water than that of a silica dispersion (see the Supporting Information). As a result, it is most likely that the predominant effect of the silica dispersion supernatant on the DOPC capacitance current peaks is from soluble Si(OH)_4_ and (HO)_3_SiO^–^. From eq 1, the available “reactive area” per 0.169 mol from 0.169 mol dm^–3^ (SiO_2_) amorphous silica particle of radius 17.5 nm dispersion is 8 × 10^5^ cm^2^. If a tetrahedrally coordinated structure of both species of dissolved SiO_2_ is assumed with a distance between the O atoms of 0.26 nm,^58^ a tetrahedral base area of ∼0.03 nm^2^ can be estimated. The 0.002 mol dm^–3^ dissolved SiO_2_ (0.012%) in equilibrium with the silica dispersion will have a “reactive area” of 3.6 × 10^5^ cm^2^ per 0.002 mol SiO_2_ if every SiO_2_ tetrahedron is close-packed on the DOPC layer surface. The adsorption of these species on the DOPC layer will account for the observed effect of the supernatant on the voltammogram of DOPC. In the silica dispersion, both the particulate and dissolved species will compete for the DOPC surface. Because the “reactive area” of the particulate silica is more than twice that of the dissolved species (8 compared with 3.5 × 10^–5^ cm^2^), the DOPC surface will be mainly occupied by adsorbed silica particles for whom the adsorption rate will be twice that of the dissolved species.

Both the observed partial reversibility of the silica nanoparticle adsorption and the almost total reversibility of the SiO_2_ dissolved species adsorption are interesting. The irreversible^59,60^ and reversible^61^ adsorption of colloids on surfaces has been reported and indeed modeled.^61^ Generally, irreversible adsorption involves chemical bond formation,^59^ whereas physical processes in adsorption such as van der Waals^61^ and H-bonding^62^ are reversible. In this study, any change in the size of the particle has no significant effect on the activation step, unlike in other studies,^60^ and the promotion of the interaction with the lipid layer by hydrated SiO_2_ species through H-bonding with the lipid polar groups is entirely consistent with near-reversible adsorption.^61^ The partial reversibility effected by silica nanoparticle adsorption is probably more because of a mechanical disturbance of the DOPC surface^15^ rather than a strong binding between particles and DOPC. A consolidation of the interaction due to binding between the hydrated SiO_2_ species and the hydrated phosphate moieties following the activation step could cause this.

This investigation has therefore established two separate observations on the adsorption of amorphous silica nanoparticles on DOPC layers. First, the heterogeneous rate constant for adsorption is constant and independent of the particle size and points to the adsorption of molecular silica species promoting the initial activation step; second, the ability of molecular SiO_2_ species to interact with the phospholipid layers is confirmed through the significant interaction of the silica dispersion supernatant with the DOPC layer. In this case, the partial reversibility of the adsorption of silica nanoparticles and the almost total reversibility of the supernatant’s interaction show that an H-bonding interaction rather than any chemical binding is a critical factor in the adsorption process. The clearest explanation of the activation step in the adsorption process is that a layer of dissolved hydrated silica molecules within a liquid structure surrounds the amorphous silica particle and that this has a thickness of 3.2 nm. It is the hydrated silica molecules within this structure, which promote the interaction with the DOPC layer through H-bonding. Because the hydrodynamic radius of the particle was used in the calculations, the “reactive layer” of hydrated SiO_2_ and associated water molecules must fall outside the hydrodynamic radius, as measured by DLS.

## Conclusions

Significantly extending a previous study,^13^ we have shown that the adsorption rate of amorphous silica nanoparticles on supported DOPC monolayers is governed by a single heterogeneous rate constant of ∼4.2 × 10^–4^ cm s^–1^. This shows that one rate-controlling step independent of the particle size controls the molecular event preceding adsorption, which leads to the conclusion that molecular SiO_2_ and hydrated more complex SiO_2_ species are the main drivers for the adsorption of the particles on the phospholipid layer. The finding is entirely compatible with (a) the occurrence of significant H-bonding between hydrated SiO_2_ species and phospholipid polar in particular phosphate groups; (b) the continuous slow dissolution of SiO_2_ into the aqueous phase leading to a well-documented hydrated silica-water interface; (c) an observed interaction of soluble SiO_2_ with the DOPC and; (d) the existence of an interfacial “reactive area” with a defined maximum “reaction layer” thickness between the silica surface and the DOPC layer. Overall, gaining an understanding regarding the adsorption rate of amorphous silica nanoparticles on to phospholipid monolayers is of great importance to elucidate their anticipated applications for silica-based nanoparticle medical applications including carrier-based drug delivery. The results of this work should also encourage spectroscopic and other studies that aim to elucidate the chemical processes that are underpinning the adsorption phenomena.

## Supporting Information Available

Additional experimental details, materials and methods, and expanded details of calculations used, providing a full underpinning of the material described in the manuscript (PDF)
