# Supplementary material for: Heterogeneous Rate Constant for Amorphous Silica Nanoparticle Adsorption on Phospholipid Monolayers
Source: Langmuir. 2022 Apr 26;38(18):5372–80. doi: 10.1021/acs.langmuir.1c03155 (PMC9097521; doi:10.1021/acs.langmuir.1c03155)
Supplement: Supplementary file 1 — la1c03155_si_001.pdf [file la1c03155_si_001.pdf]

Heterogeneous rate constant for amorphous silica nanoparticle adsorption on phospholipid monolayers

Alex Vakurov, [1] *Rik Drummond-Brydson,* [2] *Nicola William,* [1] *Didem Sanver,*[3] *Neus Bastús,*[4] *Oscar H. Moriones,*[4,5] *V. Puntes,* [4,6,7] *and Andrew L. Nelson,* [*,1]

** Corresponding author: e mail, a.l.nelson@leeds.ac.uk*

[1]School of Chemistry, University of Leeds, LS2 9JT, UK [2]School of Chemical and Process Engineering, University of Leeds, LS2 9JT, UK [3]Department of Food Engineering, Faculty of Engineering, Necmettin Erbakan University, Konya 42050, Turkey [4]Catalan Institute of Nanoscience and Nanotechnology (ICN2), CSIC and The Barcelona Institute of Science and Technology, Campus UAB, Bellaterra, 08193, Barcelona, Spain [5]Universitat Autònoma de Barcelona (UAB), Campus UAB, 08193, Bellaterra, Barcelona, Spain [6]Fundacio Hospital Universitari Vall D'Hebron - Institut De Recerca, 08035, Passeig Vall D Hebron, 119-129, Barcelona, Spain [7]ICREA, Pg. Lluıs Companys 23, 08010, Barcelona, Spain

SUPPORTING INFORMATION

1. Additional experimental details, materials, and methods

1.1. Silica adsorption rate experiments

Materials

The silica nanoparticles with the DLS hydrodynamic diameter used were as shown in Table S1. The following: Ludox SM30, LS30, AM30, and AS30 (all 30% dispersions), TM50 (50% dispersion), and CL-X (45% dispersion) in water with added NaOH (Sigma-Aldrich), Angstrom-Sphere monodispersed silica nanoparticles (ASP100) as a powder (Fiber Optics Center Inc.), and silica microspheres (SiMP50 and SiMP100, 5% in water with NaOH, Polysciences Inc.). % weights are as grams of nanoparticles per 100 cm^3 solution.

1,2-Dioleoyl-sn-glycero-3-phosphocholine (DOPC) was obtained from Avanti Polar Lipids (Alabaster, AL) and was >99% pure. PD-10 desalting columns were from GE Healthcare Bio-Science AB. All other reagents were of analytical grade and were purchased from Sigma-Aldrich.

Table S1. Silica Nanoparticle Diameters {DLS calculated as the Average of 8-20 (for Ludox Samples) or 3 (for ASP100, SiMP100, and SiMP50) Independent Measurements and the Calculated Standard Error (SE)}

Reproduced with thanks from previous article (main text, reference [13]) in ACS Langmuir

brand of nanoparticles	FiberOpt ASP100	PolySci SiMP100	PolySci SiMP50	Ludox TM50	Ludox CL-X	Ludox AM30	Ludox AS30	Ludox LS30	Ludox SM30
**nanoparticle diameter, nm (DLS by number) ± SE**	172.1 ± 2.1	100.1 ± 2.8	58.5 ± 2.2	30.1 ± 0.33	29.0 ± 0.43	19.4 ± 0.33	18.3 ± 0.28	17.8 ± 0.36	13.7 ± 0.23
**nanoparticle diameter, nm (TEM by number) ± SD (polydispersity)**	185.2 ± 8.6			30.2 ± 3.6	28.9 ± 3.3	16.5 ± 4.1		17.4 ± 3.8	10.9 ± 2.7

Methods

Electrochemical Flow System Setup: The flow system consisted of (i) a peristaltic pump (Cole-Parmer Instrument Co., cat. no. 7554-20), (ii) two universal valve-switching modules (Anachem Ltd.), (iii) a 10 dm^3 electrolyte reservoir, (iv) a 25 cm^3 sample cell, (v) two magnetic stirrers for the solutions in the electrolyte reservoir and the sample cell, and (vi) a flow cell. All parts were connected to each other by Teflon and silicon tubing. The flow cell was made from plexiglass with a silicon rubber seal. An Ag/AgCl 3.0 mol dm^{-3} KCl REF201 Red Rod reference electrode (VWR International Ltd.) was fitted into the cell, and all potentials in this article, unless otherwise stated, are quoted versus the potential of this electrode. Silicon wafer-based microfabricated Pt electrodes (Tyndall National Institute, Ireland) were inserted into the flow cell. These electrodes consisted of eight Pt discs with a diameter of 1 mm and two Pt rectangles that were 8.3 mm long and 1.8 mm wide embedded on a 28 Å~ 28 mm2 diced silicon wafer substrate possessing a surface of 0.2 µm dry silicon oxide. Each Pt area was connected to respective contact pads by a 0.5-mm-thick Pt trace interconnect that was insulated with approximately 1.5 mm of Si^3N^4 deposited by plasma-enhanced chemical vapor deposition (PECVD). The microfabricated Pt electrode was connected to a PGSTAT 30 Autolab potentiostat (Ecochemie, Utrecht, The Netherlands) interfaced to a Powerlab 4/30. signal generator (AD Instruments Ltd.) controlled by Scope software. The volume of the flow cell was 0.75 cm^3 and the internal

thickness of chamber 1 mm. The flow system was used in four different settings depending on the positions of the valves, as follows:

(1) Electrolyte was drawn from an electrolyte reservoir and discharged to waste after passing through the flow cell.

(2) Electrolyte was drawn from the electrolyte reservoir and filled the sample cell.

(3) Electrolyte was drawn from the sample cell and discharged to waste after passing through the flow cell.

(4) Electrolyte was drawn from the sample cell, passed through the flow cell, and re-entered into the sample cell by cycling.

All solutions were constantly purged with argon (Air Products) during all electrochemical experiments.

Electrode Pretreatment and RCV: The working electrodes were cleaned prior to Hg deposition in a hot solution of H_2SO_4 (Fisher Scientific) and a 30% H_2O_2 (Fluka) mixture in a ratio of approximately 3:1, respectively, and rinsed with Milli-Q 18.2 MΩ water (Millipore, U.K.) before drying under nitrogen (N_2). Hg was deposited manually on the working electrode discs using a pipette. The Pt electrodes with deposited Hg are subsequently referred to as Pt/Hg electrodes throughout the text. Rapid cyclic voltammetry (RCV) was carried out in phosphate-buffered saline (PBS) with ionic strengths (I) of 0.13 mol dm^{-3} (for 0.1 mol dm^{-3}) KCl calcined at 600°C containing 0.01 mol dm^{-3} sodium phosphate and adjusted to the required pH with HCl in the flow cell at room temperature. The PBS in the electrolyte reservoir was deaerated by purging with argon (Air Products) for 60 min before use. This electrolyte (20 cm^3) was drawn into the sample cell prior to each experiment and deaerated by purging with argon (Air Products) for 5 min. The electrochemical cell and screened cables were contained in a Faraday cage. The electrodes were connected to the Autolab potentiostat as described above. The RCV voltage excursion was from −0.4 V to potentials of between −1.125 and −3.0 V depending on the application at a scan rate of 40 V s^{-1}. Scans were repeated continuously or at an interval of 1 s between each scan.

Purification of Nanoparticle Dispersions by Gel Filtration: The PD10 desalting column was equilibrated with 10−15 cm^3 of water. A 5−10% SiO^2 nanoparticle dispersion (2 cm^3) was added to the column. After the SiO_2 nanoparticle dispersion was introduced into the column, 1 cm^3 of water was added. Then 3 cm^3 of water was added to the column in order to release 3 cm^3 of the purified nanoparticle dispersion, which was collected. The column was washed with 10−15 cm^3 of water so that it was then ready for the next purification. The purification of 14 samples of 5% Ludox SM30 silica nanoparticle dispersions on 7 different columns resulted in the production of 14 purified silica nanoparticle dispersion samples with an average concentration of 3.237 ± 0.024 (±SE) % silica. All experiments with nanoparticles were carried out after the nanoparticles were purified on the PD10 desalting column.

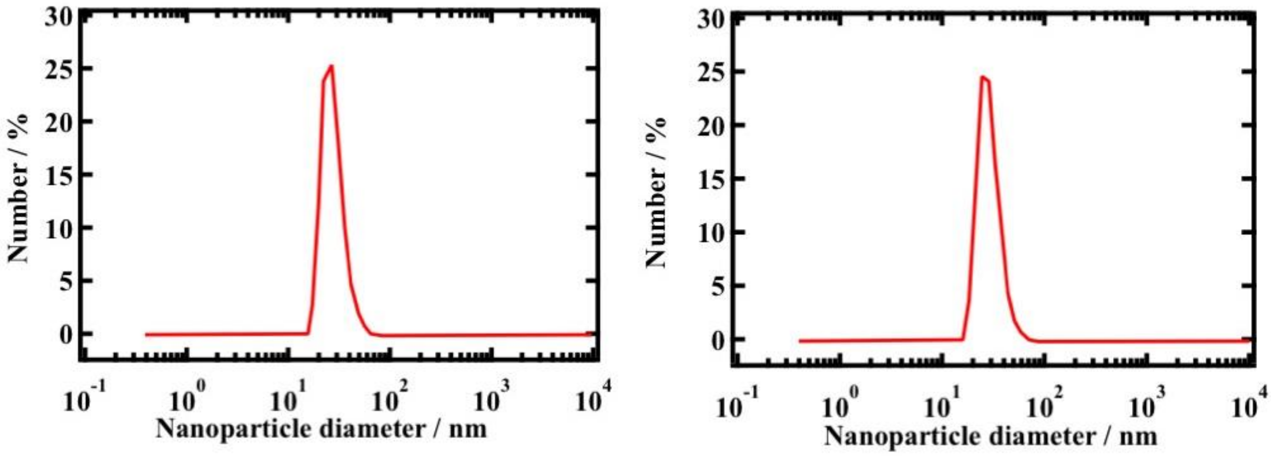

Figure S1 Dynamic light scattering **(**DLS) measurements of silica nanoparticles (radius 15.05 nm) in 0.1 mol dm^{-3} sodium phosphate buffer at pH 7 freshly sourced (left) and incubated for 2 h (right).
Reproduced with thanks from previous article (main text, reference [13]) in ACS Langmuir

Electrochemical Characterization of Interactions: The flow system was set up to valve position 1 as described above, and the flow rate was set to 1 cm^3 min^{-1}. RCV was initiated continuously at a voltage excursion from −0.4 to −3 V. A DOPC dispersion (0.2 mg cm^{-3}, 100−200 μL) was injected into the flow cell, and after 1 to 2 s when the characteristic voltammetric peaks of DOPC appeared, RCV was terminated. RCV was then initiated again with a voltage excursion from −0.4 to −1.625 V to test the quality of the DOPC monolayer as indicated by the maximum height of the voltammetric peaks (Figure 1(a)). If the quality of the DOPC monolayer was found not to be acceptable, as shown by depressed voltammetric peaks, then the coating procedure was repeated. Following successful DOPC deposition, the flow rate was set to 10 cm^3

min^{-1}. The valves were switched from position 1 to position 2 until the electrolyte filled the sample cell to 20 cm^3, and then the valves were switched to position 1 again. An aliquot of the SiO_2 nanoparticle dispersion was added to the sample cell to the required concentration using a glass micro syringe. The valves were then switched to position 3, and RCV was initiated with a voltage excursion from −0.4 to −1.125 at 40 V s−1 at a scan repetition rate of 1 scan s^{-1}. Each scan was saved and analyzed later by Excel macros. The interaction of the nanoparticles with DOPC can be followed by the plot of the voltammetric peak height with respect to the elapsed time following the initiation of the interaction. The initial rates (V) of voltammetric peak current suppression estimated from the slope of the peak 2 height versus time in region 1 of the curve were estimated at different silica particle concentrations (C_{np}). After 30 s, the valves were switched to position 4 and measurements continued for 5−20 min depending on the interaction rate. At the end of the assay, RCV was terminated and the valves were switched to position 3. When the sample cell was emptied, the valves were switched to position 2, and by the sequential switching of valves between positions 2 and 3, the system was washed. The Pt/Hg electrode was electrochemically cleaned in situ by repetitively cycling its potential from −0.4 to −3 V at 80 V s^{-1} with an electrolyte flow rate of 10 cm^3 min^{-1}, and the valves were set to position 1 for 30−60 s until the current peaks relating to absorbed material on the Hg surface disappeared.

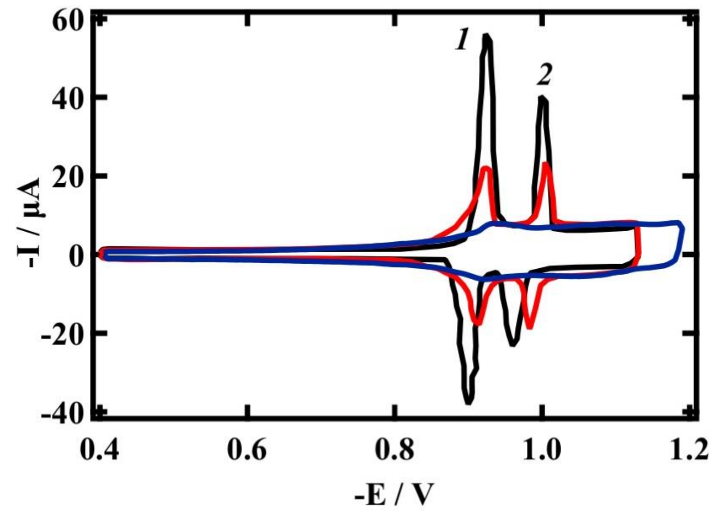

Figure S2: RCV of DOPC on a Pt/Hg electrode recorded at 40 V s^{-1} control PBS (black) and after 5 min incubation with 0.00083 mol dm^{-3} (SiO_2) 15 nm (red) (from reference [13]) and 6.75 nm (blue) radius Ludox silica dispersion in PBS at pH 7.5 (red); Capacitance current peaks 1 and 2 are marked.

Reproduced plus additional data with thanks from previous article (main text, reference [13]) in ACS Langmuir

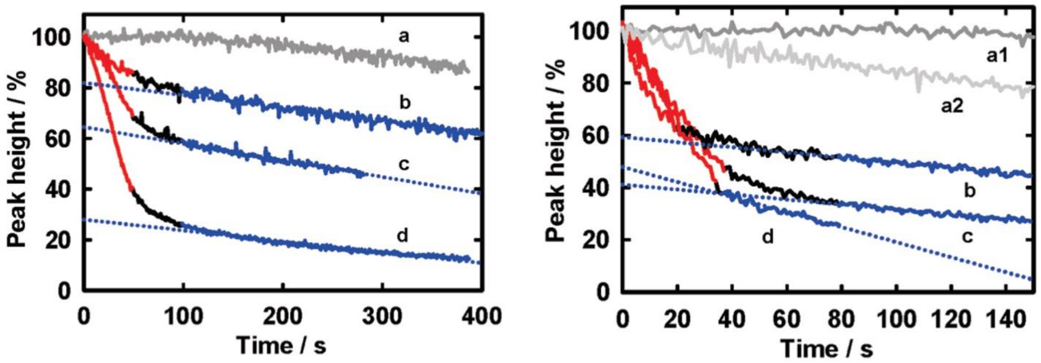

Figure S3: (left) Time curve of the RCV peak 2 height suppression expressed as a percentage of the peak height at time = 0 for DOPC-coated Pt/Hg in PBS electrolyte, I = 0.13 mol dm^{-3} at pH 7.4: (a) no SiO_2 addition, (b) +0.1% 49.91 nm (c) +0.02% 29.25 nm, and (d) +0.005% 9.15 nm radius SiO_2 showing an estimation of the residual peak height. (right) Time curve of the RCV peak 2 height suppression expressed as a percentage of the peak height at time = 0 s for DOPC-coated Pt/Hg in PBS electrolyte: (a1) I = 0.13 mol dm^{-3} at pH 7.4, (a2) I = 0.13 mol dm^{-3} at pH 4, (b–d) 15.05 nm radius SiO_2, (b) I = 0.13 mol dm^{-3} pH 7.4, (c) I = 0.13 mol dm^{-3} pH 4, and (d) I = 0.53 mol dm^{-3} pH 7.4

Reproduced with thanks from previous article (main text, reference [13]) in ACS Langmuir

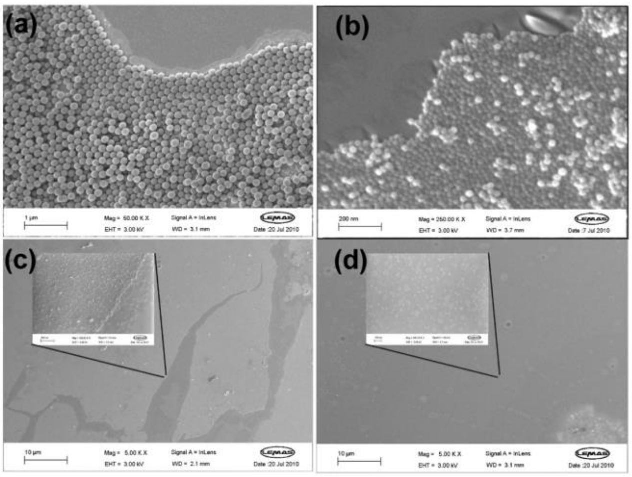

Figure S4: Scanning electron microscopy (SEM) images of (a) a DOPC-monolayer-coated Pt/Hg film electrode after incubation with 85 nm radius and (b) 15.05 radius (high magnification), (c) 6.85 nm radius SiO_2 nanoparticles (low magnification), and (d) uncoated Pt/Hg film electrode after incubation with 6.85 nm radius SiO_2 (low magnification).

Reproduced with thanks from previous article (main text, reference [13]) in ACS Langmuir

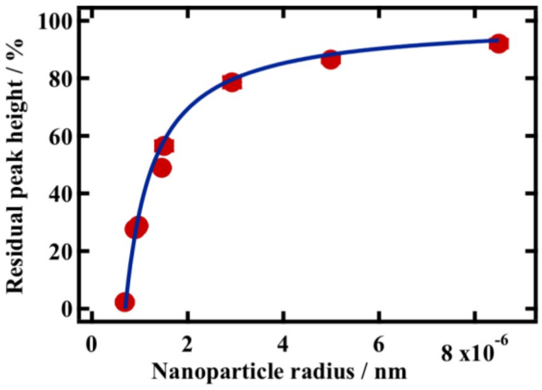

Figure S5: Plot of the RCV residual peak 2 height as a percentage of the control RCV peak 2 height versus the SiO_2 nanoparticle radius: each value was calculated as the mean height of peak 2 (±SE) from 3 to 10 experiments carried out on DOPC-coated Pt/Hg in PBS electrolyte, I = 0.13 mol dm^{-3} at pH 7.4. The blue line is fit of equation (6) of reference [13] in main text.

Reproduced with thanks from previous article (main text, reference [13]) in ACS Langmuir

1.2. Silica and dissolved silica adsorption

Materials

The description of the silica particle dispersions used is described in the main text. These were stored in MilliQ water.

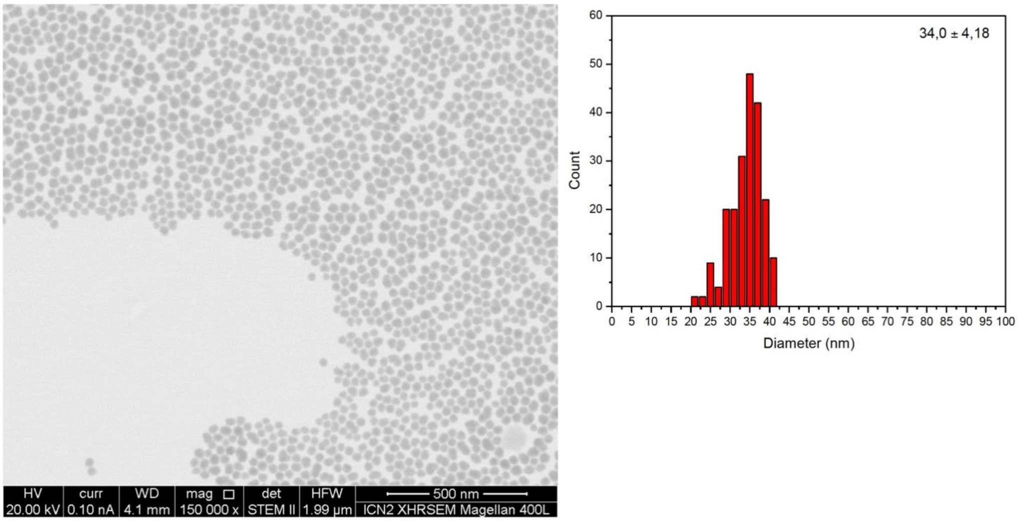

Figure S6: Transmission electron microscopy (TEM) of silica nanoparticles used in silica and dissolved silica adsorption experiments

.

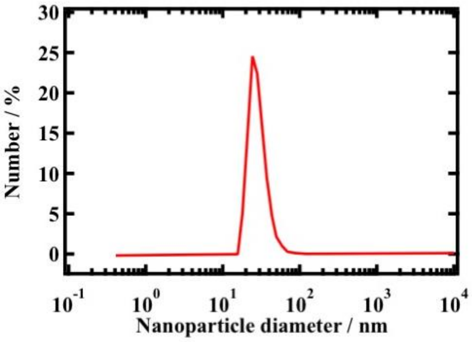

Figure S7: Dynamic light scattering (DLS) of silica nanoparticle dispersions used in silica and dissolved silica adsorption experiments

Methods

The electrochemical assay employed a similar platform and methods to that used for the adsorption rate experiments, except that a constant flow rate of 5 cm^3 min^{-1} was used in the flow cell. Furthermore, to allow

the dispersion to enter the flow cell through a valve, 2 cm^3 of dispersion and supernatant in MilliQ water respectively were injected into the line of PBS entering the flow cell using a syringe. This initially showed up as the effect of water on the current response, which causes separation and depression of the capacitance current peaks due to a transient increase in the solution resistance. Once this had passed, the effect of the nanoparticle dispersion on the phospholipid sensor element was observed. Taking account of the flow rate, the flow dynamics and the volume of dispersion injected, the maximum contact time of the silica nanoparticle dispersion and supernatant, respectively, with the DOPC sensor element was of the order of one minute.

Characterization of silica nanoparticle dispersion supernatant

The presence of silica nanoparticles in the supernatant after centrifugation was evaluated by measuring the optical density (OD) of the solutions at λ= 300 nm by UV-Vis spectrophotometry. The silica nanoparticles were centrifuged at 15000G for 20 minutes, and the supernatant was analyzed by UV-Vis. Milli-Q H2O and the silica nanoparticle dispersion prior to centrifugation were included in the analysis as negative and positive controls, respectively.

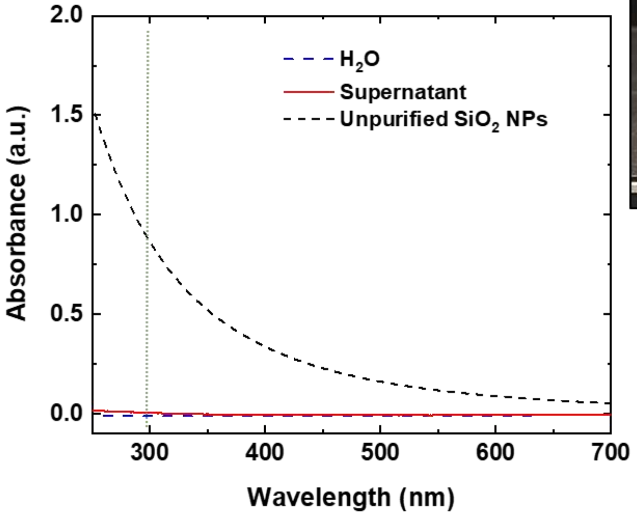

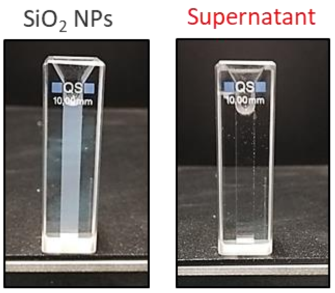

Sample	Optical density (cm^{-1})
Milli-Q water	-0,0162
Supernatant	0,0044
Unpurified SiO_2 NPs	0,8638

Figure S8: UV-vis spectrophotometery of silica nanoparticle dispersion and its supernatant compared with that of MilliQ water

Optical density (OD), also referred to as absorbance, is a measure of a material's ability to absorb light. Its value is directly proportional to the concentration of silica in the solution. As can be seen, the OD of the supernatant is almost zero. In contrast, the silica nanoparticle dispersion presents an OD of 0.8638 a.u. Results confirm the successful sedimentation of the silica nanoparticles following the centrifugation procedure.

These results were further confirmed by dynamic light scattering (DLS) by measuring the derived mean count rate (kcps). This parameter is a measure of the scattering intensity of a colloidal solution and comes from the average number of photons per second arriving at the detector divided by the attenuator factor. Higher values are direct evidence of the presence of NPs in the solution. As can be seen in Table S2, the derived mean count rate of the supernatant (37650 kcps) is ~ 4 times greater than that of Milli-Q water (8224 kcps) and ~ 11 times smaller than that of the silica nanoparticle dispersion (409900 kcps).

Table S2. Derived mean count rate of silica nanoparticle dispersion and its supernatant compared with that of MilliQ water from dynamic light scattering (DLS) measurements

Sample	Mean count rate (kcps)	Attenuator	Attenuator Factor	Derived mean count rate (kcps)
**Milli-Q water**	386.5	8	0.047	8224
**Supernatant**	154.4	6	0.0041	37650
**Silica nanoparticle dispersion**	266.4	4	0.00065	409900

Rate of silica adsorption

Figures for rates of adsorption of silica nanoparticles from dispersion are shown in Figure S9.

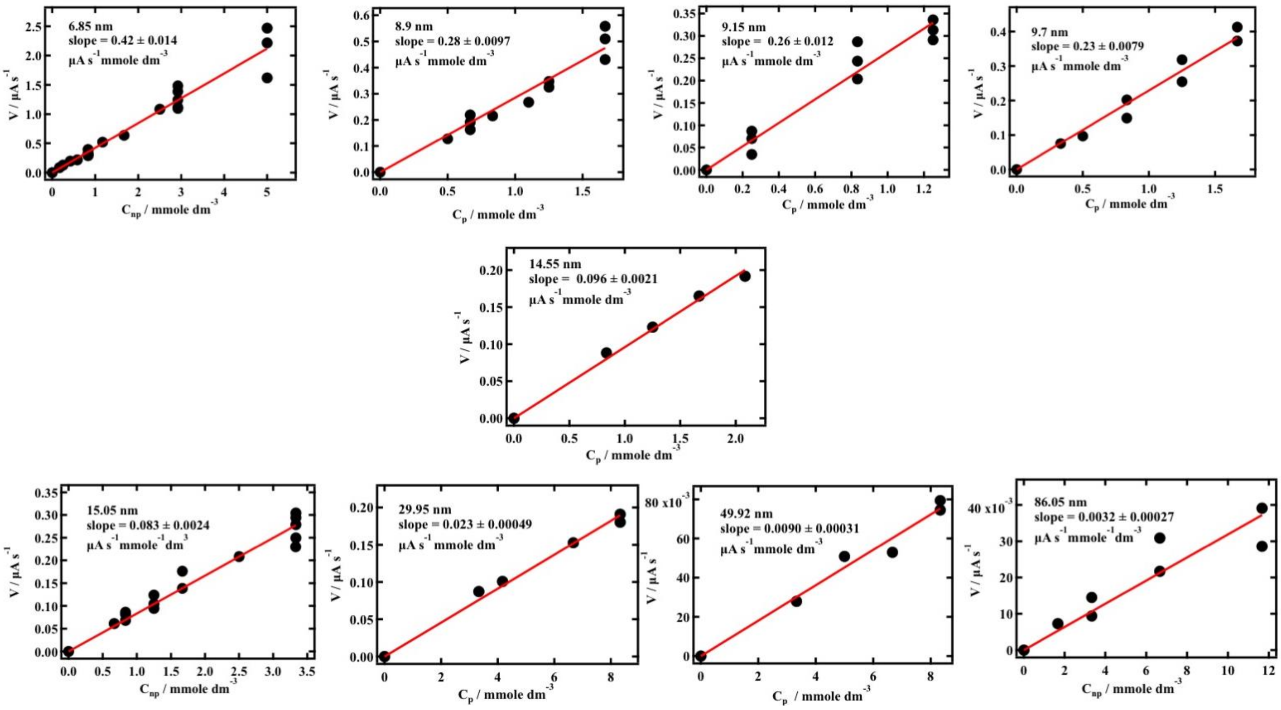

Figure S9: Plots of the rate of decrease in the height of capacitance current peak 2 height (V) versus silica nanoparticle bulk concentration as mmole dm^{-3} SiO_2 (C_{np}) derived from RCVs of DOPC on Pt/Hg exposed to silica nanoparticles in dispersion of respective radius indicated on each figure.

2. Expanded details of calculations used

Background

In the full close-packed coverage of silica nanoparticles on the DOPC layer, a single nanoparticle within a close-packed nanoparticle layer covers a planar area of two equilateral triangles fitted together to form a parallelogram where each side equals 2R and R is the nanoparticle radius in cm.

Thus, the area of the equilateral triangle is $\sqrt{3} \times R^2$, and the area of the parallelogram is $2 \times \sqrt{3} \times R^2$ or $3.46R^2$, which is approximately the area covered by one nanoparticle within a close-packed nanoparticle array. A close-packed array of these parallelograms represents the area available for nanoparticle binding to the DOPC layer.

The planar area of the nanoparticle which interacts with the DOPC is given by $\pi((R+h)^2-R^2) = \pi(2Rh + h^2)$ and is the “reactive area” in which h is termed the “reaction layer thickness” described in the main text and was found to be 3.23×10^{-7} cm from previous work.

Accordingly, the “reactive area” divided by the total planar area of a nanoparticle covering the DOPC layer is given by: $\pi(2Rh + h^2) / 3.46R^2$. When this value equals almost unity, the capacitance current peak 2 is effectively 100% depressed since the DOPC layer is totally covered by the “reactive areas” of all interacting nanoparticles, including the interstices between the close-packed particles. As a result, the residual fraction of the capacitance current peak 2 following interaction of the DOPC layer with silica nanoparticles is given by: $1-[\pi(2Rh + h^2) / 3.46R^2]$. This equation was previously validated by fitting to silica nanoparticle adsorption data using a curve-fitting procedure from which a value for h was extracted.

Note that it is physically impossible for the residual fraction of capacitance peak 2 to be negative since when the residual fraction of capacitance peak 2 approaches zero, the “reactive area” of the nanoparticle is equal to the total planar area of the nanoparticle covering the DOPC surface. For smaller particles covering the DOPC layer, the equation above remains equal to zero, but the value of h decreases as the particles get closer together.

Figure S10 (a) and (b) below shows the interaction of large and small silica nanoparticles with the DOPC layer showing the relation between the “reactive area” and the nanoparticle covering area.

(a)

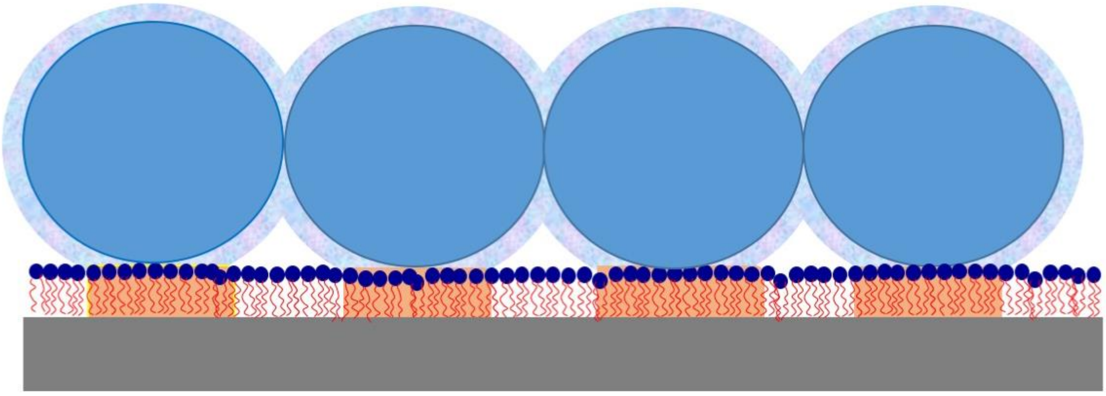

(b)

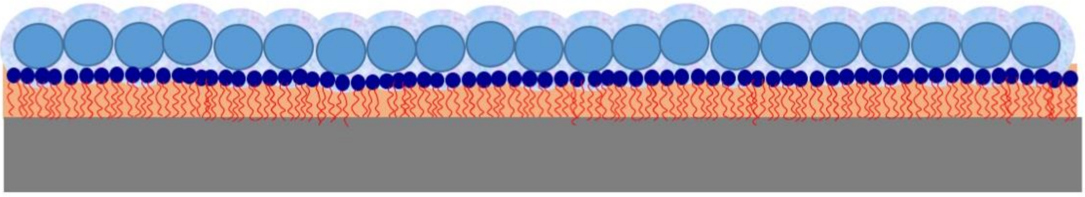

Figure S10: Adsorption of silica nanoparticles on DOPC supported monolayers with particle (blue), "reaction layer" (textured), showing "reactive area" as pink shading of monolayer: (a) large particle and (b) small particle adsorption.

Reproduced with thanks from previous article (main text, reference [13]) in ACS Langmuir

Calculation of "reactive area" of particles.

The total "reactive area" of a mmol of nanoparticles (A) is given as:

(The "reactive area" of one nanoparticle) x (total volume of nanoparticles) / (the volume of one nanoparticle).

This is given in the text as:

$A = \pi(2Rh + h^2) \times \{[(60.08 \text{ g mole}^{-1} \times 10^{-3}) / 2.196 \text{ g cm}^{-3}] / (4\pi R^3/3)\}$ Equation (S1)

where 60.08 g mole^{-1} is the molecular mass of SiO_2, h is the "reaction layer" thickness (= 3.213 x 10^{-7} cm) and 2.196 g cm^{-3} is the density of amorphous silica as defined in the text.

Calculation using "bottom up" procedure

$k_1 = k' \times 1000 / (A \times 32.1\ \mu\text{A})$ Equation (S2)

where 32.1 μA is the maximum depression of capacitance current peak 2 measured from the peak to the capacitance current baseline at that potential.

or

$k_1 = k' \times 1000 / (\pi(2Rh + h^2) \times \{[(60.08 \text{ g mole}^{-1} \times 10^{-3}) / 2.196 \text{ g cm}^{-3}]/ (4\pi R^3/3)\} \times 32.1\ \mu A$ Equation (S3)

This can be rearranged to:

$k_1 = [k' \times 10^6 \times (2.196 \text{ g cm}^{-3} / 60.08 \text{ g mole}^{-1}) \times (4\pi R^3/^3)] / [\pi(2Rh + h^2) \times 3.21\ \mu A]$ Equation (S4)

Calculations using "top down" procedure

The normalized rate (k') is converted to a heterogeneous adsorption rate constant (k_2 and k_2) by dividing it by the maximum depression of the capacitance current peak specific to that particle size (Figure 1C of reference [14])) and multiplied by the millimolar close-packed nanoparticle coverage on the DOPC surface. To carry out the calculation, the experimental maximum depression of the capacitance peak current specific to a particular particle size can be used, and the heterogeneous adsorption rate constant is written as k_2. The maximum depression of the capacitance peak current particular to a particle size can also be estimated from Equation (6) in reference [14] which fits very closely to the data and thus can also be used in the calculation of the heterogeneous adsorption rate constant and is written as k_2.

The surface area (SA) on a surface in cm^2 covered by a mmole of nanoparticles[14] is $6.02 \times 10^{20} \times 2\sqrt{3} \times R^2$ including interstices, and the reciprocal of this number is the close-packed nanoparticle coverage per cm^2.

A factor 'v' or 'v' (depending on how it is calculated) can be identified, which is the maximum depression of the capacitance peak current specific to a particle size multiplied by the SA and in the case where the maximum depression of the capacitance peak current particular to a particle size is obtained from the experiment, v, can be directly calculated. v can also be estimated from Equation (6) in reference [14] where the maximum depression of the capacitance peak specific to a particle size is $32.1\ \mu A \times \pi(2Rh + h^2) / (2 \times \sqrt{3} \times R^2)$. So $\mathrm{v} = 32.1\ \mu A \times \pi(2Rh + h^2) \times 6.02 \times 10^{20}$. This is used in the calculation of k_2. The equations for k_2 and k_2 are as follows:

$k_2 = 1000 \times C_{tr} \times k'/v$ Equation (S5)

$\mathrm{k}_2 = 1000 \times C_{tr} \times k'/\mathrm{v}$ Equation (S6)

Since *k′* as numerator is normalized to one millimole of molecules (per dm^3) and the denominator (*v* and v) is normalized to a millimole of particles (per cm^2), it is necessary to multiply the numerator by the number of SiO_2 molecules in one silica nanoparticle (C_{tr}) and, and also by 1000 to bring all units to cm, to obtain the k_2 and k_2 values which are expressed as follows:

The mass of a mole of silica nanoparticles divided by the mass of a mole of SiO_2 molecules (C_{tr}) is given by:

$C_{tr} = 6.02 \times 10^{23} \times 4/3\ \pi R^3 \times 2.196\ \mathrm{g\ cm^{-3}} / 60.08\ \mathrm{g\ mole^{-1}}$ Equation (S7)

So, the following can be written:

$\mathrm{k}_2 = (1000 \times 6.02 \times 10^{23} \times 4/3\ \pi R^3 \times 2.196\ \mathrm{g\ cm^{-3}} / 60.08\ \mathrm{g\ mole^{-1}}) \times k' / [32.1\ \mu A \times \pi(2Rh + h^2) \times 6.02 \times 10^{20}]$ Equation (S8)

or $\mathrm{k}_2 = 10^6 \times 4/3\ \pi R^3 \times (2.196\ \mathrm{g\ cm^{-3}} / 60.08\ \mathrm{g\ mole^{-1}}) \times k' / [32.1\ \mu A \times \pi(2Rh + h^2)]$ Equation (S9)

It is noted that Equation (S9) is identical to Equation (S4).
